# Effects of gravity, microgravity or microgravity simulation on early mouse embryogenesis: A review of the first two space embryo studies

**DOI:** 10.1016/j.mbm.2024.100081

**Published:** 2024-07-20

**Authors:** Douglas M. Ruden, Daniel A. Rappolee

**Affiliations:** a C. S. Mott Center for Human Health and Development, Department of Obstetrics and Gynecology, Wayne State University, Detroit, MI, United States; b Institute of Environmental Health Sciences, Wayne State University, Detroit, MI, United States

**Keywords:** Microgravity, Clinostat, International space station (ISS), Embryogenesis

## Abstract

Many simulated micro-gravity (micro-G) experiments on earth suggest that micro-G conditions are not compatible with early mammalian embryo development. Recently, the first two “space embryo” studies have been published showing that early mouse embryo development can occur in real microgravity (real micro-G) conditions in orbit. In the first of these studies, published in 2020, Lei and collaborators developed automated mini-incubator (AMI) devices for mouse embryos facilitating cultivation, microscopic observation, and fixation^1^. Within these AMI apparatuses, 3400 non-frozen 2-cell embryos were launched in a recoverable satellite, experiencing sustained microgravity (∼0.001G) for 64 ​h post-orbit before fixation in space and recovery on earth. In a subsequent study, in 2023, Wakayama and colleagues^2^ devised Embryo Thawing and Culturing (ETC) devices, enabling manual thawing, cultivation, and fixation of frozen 2-cell mouse embryos by a trained astronaut aboard the International Space Station (ISS). Within the ETCs, a total of 720 2-cell mouse embryos underwent thawing and cultivation for 4 days on the ISS, subject to either microgravity (n ​= ​360) and simulated-1G (n ​= ​360) conditions. The primary findings from both space embryo experiments indicate that mouse embryos can progress through embryogenesis from the 2-cell stage to the blastocyst stage under real micro-G conditions with few defects. Collectively, these studies propose the potential for mammalian reproduction under real micro-G conditions, challenging earlier simulated micro-G research suggesting otherwise.

## 1 Introduction

*This is Major Tom to Ground Control …*

*And I'm floating in a most peculiar way.*

David Bowie, “*Space Oddity*” lyrics^3^

The ability of embryos to develop in space is necessary to ensure the long-term survival of humans in space. In David Bowie's song quoted above, the term “Ground Control” refers to the flight controllers on earth who monitor his progress in space. However, for developmental biologists, “ground control” refers to experiments conducted on Earth under standard gravity conditions (1G). This song has become an anthem for the hazards of space on human health and the future of humanity in space. The popularity of this song among space enthusiasts is at least in part because Astronaut Chris Hadfield delivered a memorable and creatively adapted rendition of “Space Oddity,” in 2013, while aboard the International Space Station (ISS) (https://www.youtube.com/watch?v=KaOC9danxNo).

The purpose of this review is to compare and contrast the first two space embryo studies with each other and with simulated micro-G studies of early embryos on earth. The first of these space embryo studies was done by Lei et al. and published in 2020 using and automated mini-incubator (AMI) on a recoverable satellite.^1^ The second space embryo study was by Wakayama et al. in 2023, using an Embryo Thawing and Culturing (ETC) device that required hands-on administration by an astronaut aboard the ISS.^2^ Prior to publishing the second space embryo experiments by Wakayama et al.,^2^ a review comparing the first space embryo study by Lei et al.^1^ with the earlier simulated micro-G experiments with mouse embryonic stem cells (mESCs) was published in 2021 by Li et al..^4^ In their review, Li et al.^4^ argued that both real and simulated micro-G experiments done with mESCs showed much more severe effects on development of these cells that real micro-G experiments done with mouse embryos in recoverable satellites.^4^ To distinguish our review from the review by Li et al.,^4^ we will primarily focus on comparing and contrasting the first two real micro-G space embryo experiments, after first briefly reviewing historical space embryology studies with pregnant organisms in orbit.

The investigation into the impact of gravity on early mammalian embryogenesis is needed to ensure the long-term survival of humans in space. This due to gravity's poorly understood role in exerting a mechanical force crucial for proper reproduction and mammalian development. In a paper we wrote in 2018,^5^ we reviewed experiments done on earth in micro-G simulators that work by using specialized centrifuges called clinostats or tilting-type devises that cause the embryos to continuously fall. However, these simulated micro-G devices do not fully replicate true space conditions because they have the added stress induced by hydrostatic shear caused by the centrifugation process itself. Such hydrostatic shear stress is not a concern in the space embryo experiments, but other issues such as gravity-dependent convection of fluids and changes in the relative buoyancy of cells in the blastocoelic fluid also need to be addressed, as we noted in our earlier review.^5^

Since 1965, eight distinct animal species have been subjected to experiments aboard autonomous recoverable satellites, manned space vehicles, or stations to explore their reproductive and early developmental processes. This review provides a brief overview of the animals employed in studies focusing on reproduction and early development. However, the primary emphasis here lies in comparing the first two space embryo studies that delve into mouse embryogenesis, specifically examining development from the 2-cell stage to the blastocyst stage under real micro-G conditions.^1^^,^^2^

## 2 Animals used in developmental biology and reproduction studies in space

**Fig. 1:**
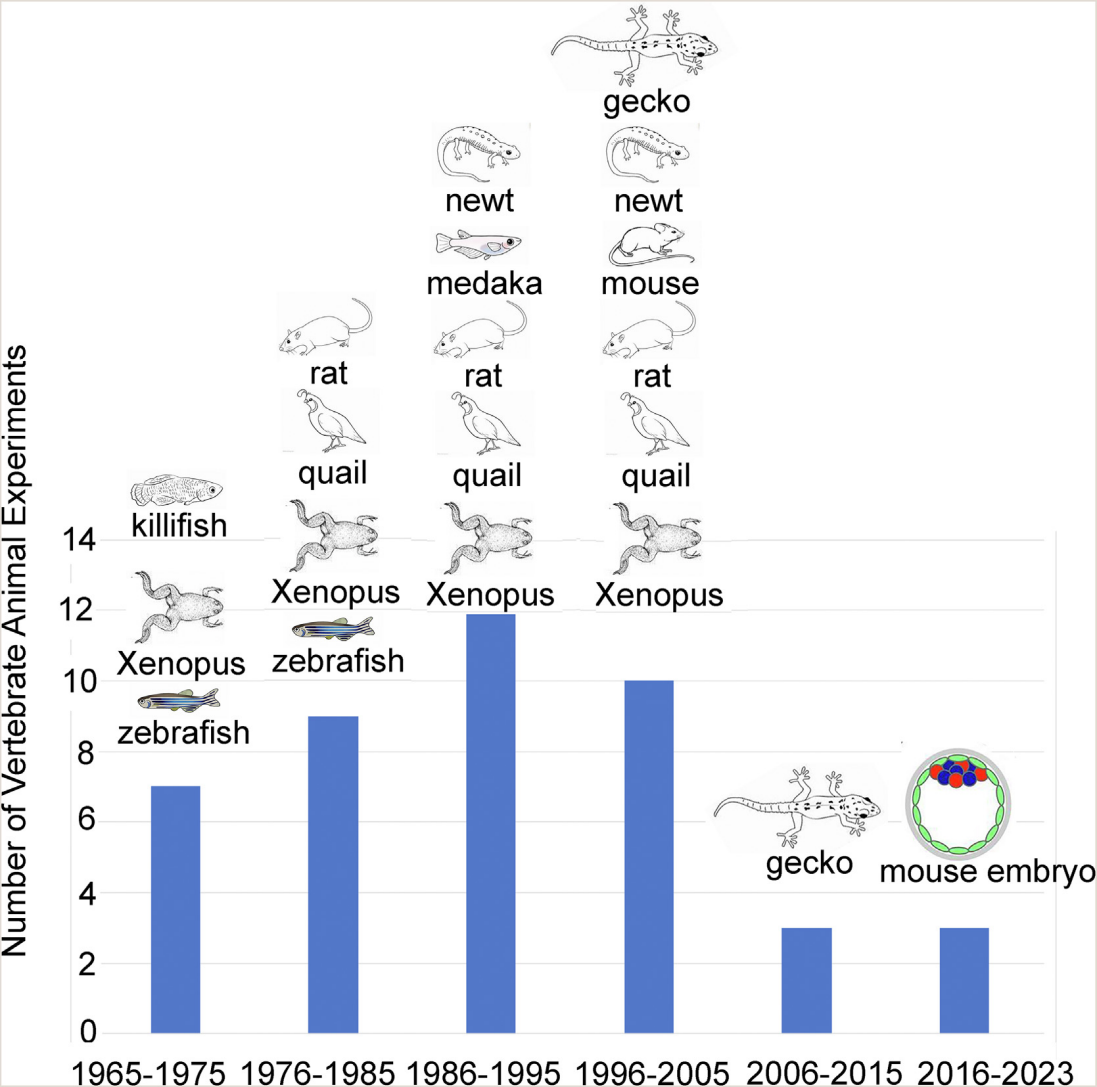
Vertebrate Animals Used for Reproduction and Developmental Biology Studies in Space. The data is from Proshchina et al. (Table 1) and is represented here in graphical form.

In 2021, Proschina and colleagues carried out a thorough review on animals subjected to microgravity in space conditions.^6^ We have condensed the information from their Table 1 into a graphical format, including two additional studies on mouse embryos conducted between 2006 and 2013^7^^,^^8^ (Fig. 1). From 1965 to 1975, the animals employed in studies on reproduction and early development included Xenopus (African clawed frog), zebrafish, and killifish. Rats and quail were added to the research from 1976 to 1985, followed by the inclusion of Medaka fish and newts from 1986 to 1995. Geckos were introduced into the studies from 1996 to 2005. Post-2005, there was a notable decline in animal investigations related to reproductive biology and development. From 2006 to 2015, the focus shifted primarily to geckos, and the subsequent period from 2016 to 2023 saw a limited number of studies, specifically the two mouse space embryo experiments discussed in the following sections.^1^^,^^2^

**Table 1:** Comparisons between Lei et al.^1^ and Wakayma et al.^2^ experiments.

Experiments and Limitations	Lei et al.^1^	Wakayama et al.^2^
Ground Control (1G)	Done in China	Done in Japan
Real micro-G	Recoverable satellite	International Space Station (ISS)
Simulated 1G	Not done on recoverable satellite in space	Done with centrifuge in incubator on ISS
Simulated micro-G	Done with clinostat on earth	Done with clinostat on earth
Radiation Exposure (1G) (on earth)	Only done on earth with radiation source (0.15, 0.5, and 2.0 ​mGy/day)	Not done
γH2AX staining for double strand DNA (dsDNA) breaks	Space embryos showed increase in dsDNA breaks compared with ground control	No dsDNA breaks seen
Thawing 2-cell embryos	Not needed (fresh 2-cell embryos loaded onto recoverable satellite)	Frozen 2-cell embryos thawed in space on ISS
Time-Lapse Microscopy (micro-G)	The micro-incubator had an attached microscope with poor resolution	The ISS did not have a microscope to study embryo development
Vibrations During Liftoff	Fresh 2-cell embryos subjected to strong vibrations and high-G during liftoff	Cryopreservation presumably protected against vibration damage.
Oct4 and Nanog Staining	Cells visualized with both anti-Oct4 (ICM) and anti-Nanog (epiblast) antibodies	Inner cell mass (ICM) cells visualized with anti-Nanog antibodies
Ctx4 Staining trophectoderm (TE)	Trophectoderm (TE) cells visualized with Ctx4 antibodies.	TE cells visualized with Ctx4 antibodies.
Bacterial Contamination (DNA sequencing)	Not done	Bacterial contamination seen visually (cloudy media) and by DNA sequencing
RNA-sequencing blastocysts	Not done	Transcriptome wide studies of entire embryos with next generation cDNA sequencing
Bisulfite-sequencing blastocysts	Saw decrease in DNA methylation in both real micro-G in space and 1G radiation experiments on earth.	Not done
Twinning of embryos	Not observed	Higher frequence of twinning in real micro-G conditions

In the review by Proschina et al.,^6^ they provide a summary and analysis of the effects of spaceflight conditions on the reproduction and early development of vertebrates. While certain developmental stages have been demonstrated to occur in space, it is noteworthy that, as of now, no mammalian (or vertebrate) animal has completed its entire life cycle—from conception to adulthood—while in space. Preliminary findings shed light on how the space environment influences critical phases of reproduction and development, including fertilization, embryogenesis, pregnancy, birth, postnatal maturation, and parental care. The Proschina et al.^6^ review is limited because it does not include Laika, the first and most famous animal to orbit the earth,^9^ or the many chimpanzees and monkeys that the USA and the Soviet Space Programs sent to space because these animals were not used for studying reproduction or early development. Rather, these early studies were to test engineering principles and survival of the test animals.^10^

The only mammals sent to space for reproduction and early development studies were rats starting in 1986,^7^^,^^8^ and mice starting in 2003.^11^ These rat and mouse experiments were primarily designed to study the effects of muscle adaptations for several days under micro-G conditions. In the year 2000, Ronco and Alberts conducted research in rats to examine the impact of spaceflight on mid to late pregnancy and birth at 1G, utilizing two distinct space shuttle missions for their study.^12^ The occurrences related to parturition, including labor, delivery, maternal care, and the initiation of nursing, were examined in female Norway rats (*Rattus norvegicus*) subjected to either 11 or 9-day-long space flights, commencing at the approximate midpoint of their pregnancies.

Each space shuttle mission concluded on the 20th day of the rats' pregnancies, just 48 ​± ​72 ​h prior to parturition. Analysis of the parturition process revealed that, in comparison to ground controls, the dams from the space flight group exhibited twice the number of lordosis contractions, which are the predominant type of labor contractions in rats. However, other parameters such as the duration of labor, fetal wastage, the number of neonates born, neonatal birth weights, and maternal care during parturition, including the onset of nursing, were found to be similar in both the flight and ground control dams. These findings suggest that, except for the increased occurrence of lordosis contractions, the qualitative and quantitative aspects of mammalian pregnancy and parturition remain largely unaffected after exposure to space flight during pregnancy.

## 3 The effects of microgravity on mouse embryo development

**Fig. 2:**
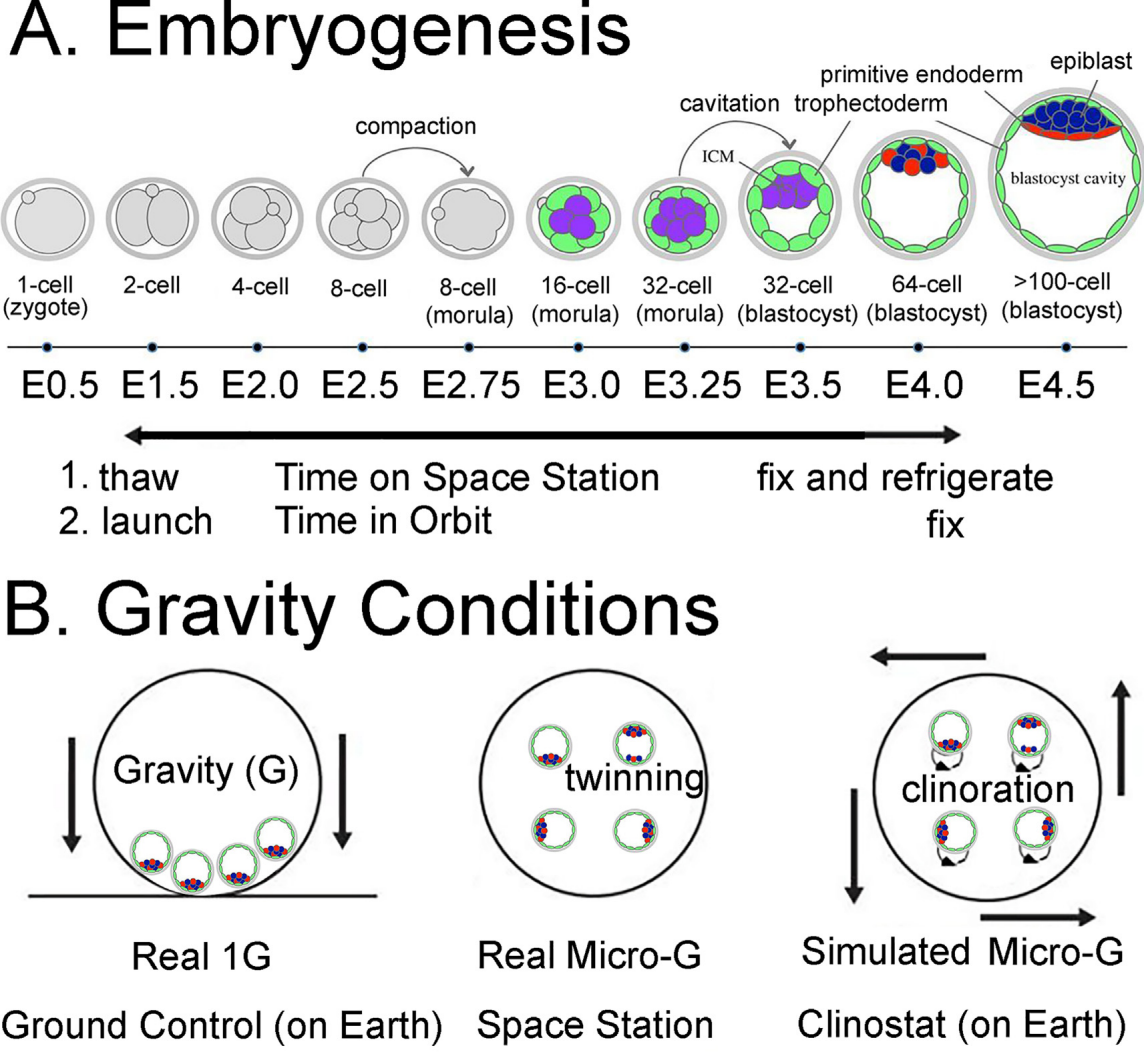
Mouse Embryo Experiments in Space. A. The illustration depicts mouse embryogenesis progressing from the 1-cell (zygote) stage to the >100-cell (blastocyst) stage. Both space embryo experiments conducted covered the developmental span from the 2-cell stage to the blastocyst stage, occurring approximately four days later. **B.** Gravity experiments were conducted in both studies, encompassing Ground Control experiments conducted on Earth (Real 1G experiments, left), Real Microgravity (Micro-G) experiments conducted on the retrievable satellite in Lei et al. and with the International Space Station (ISS) in Wakayama et al. Both studies also integrated Simulated Microgravity experiments using Clinostat-like devices on Earth. Notably, the Real Micro-G experiments exhibited an increase in twinning, denoted by two loci of Nanog-staining cells (inner cell mass (ICM); red and blue circles). The trophectoderm (TE) cells surrounding each embryo are represented by green circles. Additionally, the Clinostat experiments induced clinorotation, involving the spinning of embryos in culture, resulting in shear stress on the TE cells, potentially leading to a decrease in their numbers.

In 2018, before the two space embryo experiments that is the focus of this review, we wrote an earlier review, “Effects of Gravity, Microgravity or Microgravity Simulation on Early Mammalian Development”.^5^ Here, we update the first review by describing two recent space embryo experiments that involved the cultivation of hundreds of 2-cell mouse embryos for a ∼4-day period until many of them reached the blastocyst stage, aiming to investigate the impact of real micro-G on early embryonic development.^1^^,^^2^ We provide a comparative analysis of the two experimental approaches: one conducted on an unmanned recoverable satellite and the other on the International Space Station (ISS). Additionally, we compare the findings of the first two space embryo studies (Fig. 2; Table 1).

**Fig. 3:**
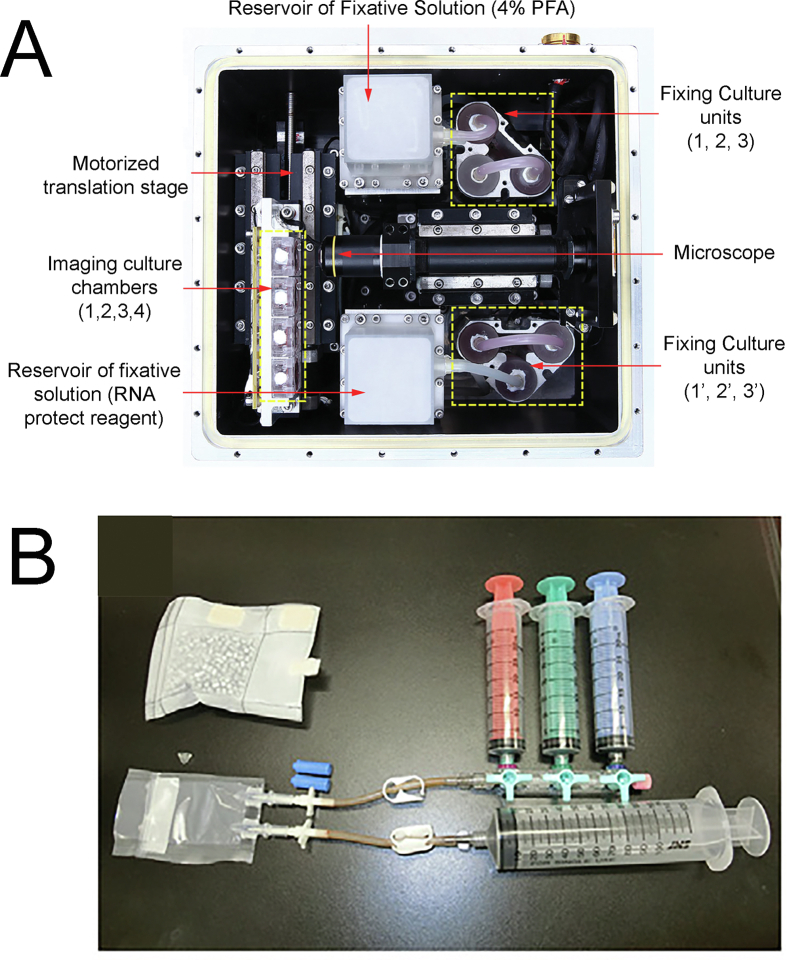
Embryo Culture Devices for Space. **A**, Automated Mini-Incubator (AMI) device created by Lei et al. The device contained a Motorized translation stage to move the Imaging culture chambers (1,2,3,4) in front of the Microscope. There was also a Reservoir for Fixative Solution (4% PFA) containing 4% paraformaldehyde (top), and a Reservoir of fixative solution (RNA protect reagent) for the whole-embryo RNA-seq experiments conducted upon return to Earth. **B**. Embryo Thawing and Culturing (ETC) device created by Wakayama et al. The ETC was connected to three syringes for solution replacement (red, green, and blue) and a syringe for waste solutions (bottom).

In the first space embryo experiments by Lei et al.,^1^ published in 2020, the research was centered on the developmental stages of mouse preimplantation embryos during the spaceflight of China's SJ-10 recoverable satellite in 2016. To facilitate this exploration, a specially designed Automated Mini-Incubator (AMI) was created, equipped with programmable controls for temperature, automated micrography, and sample fixation of the mouse embryos (see Fig. 3A). The study, for the first time, definitively established that preimplantation mouse embryos can progress through development in a space environment.

Throughout the spaceflight, in the Lei et al.^1^ experiments, the embryos underwent successive cell divisions, ultimately adopting blastocoel morphology. However, there was a conspicuous decrease in the rate of blastocyst formation and the quality of blastocysts. Additionally, the cells within the embryos manifested significant double-strand DNA breaks, as determined by γH2AX staining, and the genomes of the blastocysts developed in space exhibited global hypomethylation with a distinct set of differentially methylated regions (DMRs), as determined by whole-genome bisulfite sequencing. They also saw a significant increase in single-strand DNA breaks in the space embryos by staining them with anti-XRCC1 antibodies.^1^

Importantly, in the Lei et al. experiments,^1^ the developmental abnormalities, DNA damage, and epigenetic irregularities observed could be largely replicated through ground-based low-dose radiation treatment (2 ​mGy for 64 ​h), suggesting that cosmic radiation is a primary contributor to these developmental defects. This research enhances our comprehension of embryonic development in space and underscores the potential risks posed by long-term exposure to extremely low-dose radiation on mammalian reproduction.

In a subsequent investigation led by Wakayama et al.,^2^ the study homed in on mouse preimplantation embryonic development aboard the International Space Station (ISS). Meticulous planning characterized the experiments on the ISS, addressing the challenges inherent in manned microgravity (micro-G) experiments. For the execution of this experiment, a novel embryo thawing and culturing (ETC) device was devised. This unit facilitated the thawing and cultivation of frozen mouse 2-cell embryos on the ISS without direct astronaut contact with the embryos (see Fig. 3B). This device also required minimal astronaut training and was thoroughly pilot tested by untrained technicians on earth.^2^

In contrast to the unmanned recoverable satellite employed in Lei et al.'s study,^1^ the Wakayama et al.^2^ investigation faced several limitations due to safety considerations mandated for a manned space station. Notably, the use of toxicants, including the common chemical fixation agent 4% paraformaldehyde (PFA), is restricted on the International Space Station (ISS). Consequently, the researchers explored the viability of using 0.99% PFA, an alternative approved for use on the ISS, for the fixation of blastocysts. Following fixation with 0.99% PFA, the blastocysts were refrigerated for approximately one month before being returned to Earth.^2^

In both instances involving the launch of living embryos, the embryos were subjected to intense vibrations and hypergravity during the launch. To mitigate this effect, Wakayama et al.^2^ introduced the Embryo Thawing and Culturing (ETC) device designed for utilization on the International Space Station (ISS) by astronauts (refer to Fig. 3B). Notably, the ETC stands out as the first device enabling the entire process to be conducted on the ISS, even by astronauts without specialized training. It is worth mentioning that the Lei et al. study^1^ employed non-frozen 2-cell embryos, potentially contributing to variations between the two studies, partly due to the impact of vibrations and hypergravity on these embryos.

While the ETC device demonstrated effectiveness on Earth, a significant number of embryos within the device faced mortality or halted development at the two-cell stage when deployed on the International Space Station (ISS). The authors attribute this outcome to the cryoprotectant agent, which proved toxic to the embryos, not being adequately removed from the ETCs, leading to cytotoxicity.^2^ Nevertheless, as some embryos progressed to the blastocyst stage, it is speculated that the absence of liquid convection might have created distinct culture areas within the same ETC—some with persisting cryoprotectant and others with successful removal. Notably, since cryoprotectants were not utilized by Lei et al.^1^ in the retrievable satellite experiment, this concern did not arise. However, the lack of convection could impact the oxygenation of embryos in both sets of microgravity experiments.

In the experiments conducted on the International Space Station (ISS) by Wakayama et al.,^2^ despite the embryos demonstrating the ability to progress into blastocysts, a significant challenge arose due to bacterial contamination affecting all ETCs. The identification of bacterial contamination in all ETCs was accomplished through sequencing bacterial DNA in the embryo cultures. This contamination likely played a substantial role in the diminished rate of blastocyst development. Recognizing these outcomes as potential limitations, the researchers implemented proactive measures to address them, such as having the astronaut shake the ETCs during solution exchange. Although bacterial contamination was not explicitly acknowledged as an issue in the experiments conducted by Lei et al.,^1^ it remains unclear whether a comprehensive investigation, such as sequencing experiments to detect bacterial DNA, was conducted in that study.

The experiments conducted on the International Space Station (ISS) by Wakayama et al.^2^ presented unexpected complexities. Specifically, maintaining sterile conditions posed a significant challenge, as alcohol disinfection on the bench was prohibited on the ISS. Despite these challenging conditions, the astronauts managed to successfully generate blastocysts through the project. Upon the return of the fixed blastocysts to Earth, the authors were able to analyze them. In contrast, the retrievable satellite experiments conducted by Lei et al.^1^ introduced a different set of intricacies. Their device needed to operate fully autonomously, without the ability of a trained specialist to troubleshoot any unforeseen issues.

## 4 Discussion

While both space embryo experiments, the first done on recoverable satellites and the second on the ISS, revealed that mouse embryos can progress from 2-cell embryos to blastocysts in microgravity (micro-G), there are notable disparities in interpretations that warrant resolution (Table 1). In the initial space embryo experiments, Lei et al.^1^ raised the prospect that space radiation might have influenced embryonic development in orbit. Cultivating embryos at an orbital altitude of 252 ​km, they observed that exposure to space radiation (0.15 ​mGy/d, 64 ​h of incubation; <0.4 ​mGy in total) led to a reduction in the rate of blastocyst development, accompanied by severe DNA damage (γH2AX and XRCC1 staining) and epigenetic abnormalities. Intriguingly, despite the test embryos being subjected to a ∼10-fold higher dose of space radiation (4.29 ​mGy) on the ISS, the quality of the blastocysts developed under both space and ground control (1G) conditions was found to be comparable in the study by Wakayama et al.^2^

While the complexity of space radiation could not be entirely replicated in the ground-based irradiation experiments, the ground studies revealed the extreme radiosensitivity of early-stage embryos. This is consistent with mouse embryonic stem cell (mESC) experiments done in our laboratories that showed that these cells have extreme sensitivity to gamma irradiation and extensive changes in DNA methylation, such as in DNA repair genes.^13^ More experiments are needed both on the ground and in space to resolve the issue of precisely what doses and what kinds of space radiation is affecting development and DNA methylation.

Furthermore, the lethal dose 50 (LD_50_) for two-cell mouse embryos is documented to be 300 ​mGy or higher.^14^^,^^15^ There is skepticism regarding whether the doses of space radiation employed, specifically 0.4 ​mGy (utilized by Lei et al.^1^) or 4.29 ​mGy (utilized by Wakayama et al.^2^), could account for the observed poor survival and development rates in the space experiment groups.

Previous ground-based simulated microgravity experiments, conducted by us and others using a clinostat, have reported developmental arrest of embryos and a reduction in the cell number of trophectoderm (TE) cells. This suggests that microgravity may have an adverse impact on early embryo development.^16^, ^17^, ^18^ Clinostats simulate microgravity on Earth by subjecting embryos to continuous falling, creating a condition termed “Synthetic Micro-G" (see Fig. 2B). However, the rotation of clinostats induces a corresponding rotation of embryos, a phenomenon referred to as “clinorotation,” which can lead to shear stress on the embryos.

For the experiments that were done in orbit, the two space embryo papers studied mouse embryogenesis in the absence of shear stress generated by all existing micro-G simulators. For example, the Random Positioning Machine (RPM) generates shear stress, with the maximum near the walls of the vessel. Therefore, embryos have the most shear stress when near the walls of the vessel. The clinostat also generates some shear stress but less than the RPM.^19^ An epithelium grown near the walls of the RPM experiences enough shear to be extruded from the surface of the epithelium in a first step, to form a spheroid during the second step.^20^ This is important because the outer cells of the blastocyst make up the placental trophectoderm epithelium. This trophectoderm is protected by a zone pellucida which is an extracellular matrix create during oogenesis in the ovary, and is an acellular gel of about 4% protein 21, so the shear is reduced and the outer cells somewhat protected from extrusion.

A Rotating Wall Vessel (RWV) simulates micro-G when its axis is perpendicular to the earth's gravity (“Ferris wheel” mode) but has little micro-G simulation when its axis of rotation is parallel to earth's gravity (“carousel” mode) but still generates shear stress. The isolate shear stress or carousel was measured for its effects on the blastocyst and it is toxic, mediated by SAPK, after 12 ​h.^22^ These shear effects of the RWV have been reviewed previously.^5^ However outer cells for the blastocyst do activate cfos, a shear stress marker, as well as stress activated protein kinase (SAPK). And as the blastocyst hatches out of its zona pellucida using proteases that dissolve the zona pellucida, it becomes much more sensitive to shear stress and activates SAPK more quickly and to higher levels during embryo pipetting.^23^ The severity of shear stress, and micro-G simulation in terms magnitude of SAPK activation was not trivial.^17^^,^^22^ It is as high as the peak levels induced by positive SAPK control hyperosmotic stress at its peak inductive exposure.^24^

The clinostat generates less shear stress than the RPM,^25^ and the use of shear stressed induced bioluminescence of dinoflagellates to quantitate shear could be an important control for any type of microgravity simulation using mammalian cells or embryos. But the clinostat can still create highly significant adverse effects that are not due to micro-G simulation.^26^ In this analysis the authors conclude that, like the use of Ferris wheel and carousel modes to dissect shear stress from microgravity simulation above during RWV use, the clinostat must be used in modes and controls which emphasize and de-emphasize shear to isolate and understand the relative role of micro-G simulation in the mechanisms and their roles in biological outcomes.

Wakayama et al.^2^ convincingly demonstrate the feasibility of differentiation into Inner Cell Mass (ICM) and Trophoblast (TE) cells under authentic microgravity conditions on the International Space Station (ISS) in the absence of the shear stress observed on Earth based micro-G simulations. Similarly, Lei et al.,^1^ who also conducted orbital experiments, observed a comparable outcome. The adverse impact on embryonic development observed in the clinostat experiment likely stemmed from the rotational culture induced by the clinostat, rather than being solely attributable to the effects of microgravity. Notably, when embryos were cultured on the ISS, the artificial 1G generated by rotation resulted in a lower number of TE cells compared to microgravity conditions. This aligns with findings from our laboratory indicating that Trophoblast Stem Cell (TSC), which correspond to TE cells when grown in culture, are considerably more sensitive to stress than Embryonic Stem Cells (ESC), which correspond to ICM cells when grown in culture.^27^

Maitre et al.^28^ highlighted that the interplay between cell positioning and fate specification, facilitated by contractility, enables blastomeres to anticipate their final position and initiate differentiation accordingly. If Earth's gravity were to influence the positional alignment and introduce even slight tension to embryos, it could potentially impact cell positioning and initial differentiation under microgravity conditions. Intriguingly, when embryos were cultured in microgravity on the International Space Station (ISS), blastocysts with ectopic expression of NANOG cells were identified in 3 out of 12 embryos (25%), representing a higher incidence rate compared to artificial 1G (0%) or ground control (1G) embryos (7%). Notably, Lei et al.^1^ did not observe a higher incidence of twinning in their study, but it is likely that they did not as thoroughly investigate the twinning effect. We believe that the twinning effect is an intriguing finding in the ISS experiments and warrants further investigation.

Wakayama et al.'s^2^ ground-based experiments suggest that Inner Cell Mass (ICM) cells are denser than other cells and tend to sink to the bottom of the blastocyst cavity. In the microgravity environment of the International Space Station (ISS), where gravity is nearly zero, ICM cells would not exhibit the same sinking or clustering behavior in one specific location within the blastocyst cavity. While this study does not clearly establish whether the ectopically expressed NANOG-positive cells were indeed ICM cells, if they were, it is conceivable that they might show less tendency to cluster in microgravity.

In instances where the ICM cell is divided into two pieces, identical twins are formed, sharing the same placenta. Armadillo reproduction involves the ICM cell splitting into four, resulting in the birth of monozygotic quadruplets. However, it is crucial to acknowledge that the separation of the ICM cell is a rare event in mammalian reproduction. In such occurrences, it imposes an increased burden on both fetuses and mothers, elevating the risk of miscarriage, particularly in species that typically have single-gestation pregnancies. Given that mammals evolved in a 1G environment on Earth, it is plausible that they have adapted to rely on Earth's gravity for ensuring safe pregnancies and deliveries.

Microgravity during space flight has been documented to induce subtle abnormalities in the fertilization and embryonic development of sea urchins and amphibians.^29^, ^30^, ^31^ However, in the case of Medaka fish, mating, fertilization, and hatching during an orbital experiment resulted in offspring with apparently normal ovaries and fertility.^32^ Furthermore, space flight in mice during mid-to-late gestation demonstrated modest effects on the birth rate, litter size, birth weights, and neonatal mortality.^12^^,^^33^ Drawing from these findings and the results of the two space embryo experiments discussed, it appears that mammalian reproduction in space might be feasible, albeit with some potential impact. Regrettably, the number of blastocysts obtained from the two space embryo experiments was not abundant, and as of now, no one has confirmed the impact on offspring, as there have not yet been any attempts to produce offspring from embryos developed in space.

## 5 Recommended improvements in future space embryo studies

The two space embryo experiments done by Lei et al.^1^ and Wakayama et al.^2^ show two approaches to conducting these studies. Lei et al.^1^ approached the problem by having every step in analyzing the space embryos automated after loading the freshly collected 2-cell embryos onto the recoverable satellite and fixing them automatically in space when some of them potentially reached the blastocyst stage if development occurred normally. Wakayama et al.^2^ approached the problem by freezing the 2-cell embryos and having an astronaut thaw them and incubate them in media until they were expected to reach blastocyst stage. Here we discuss advantages and disadvantages to both approaches and suggest improvements.

**Fig. 4:**
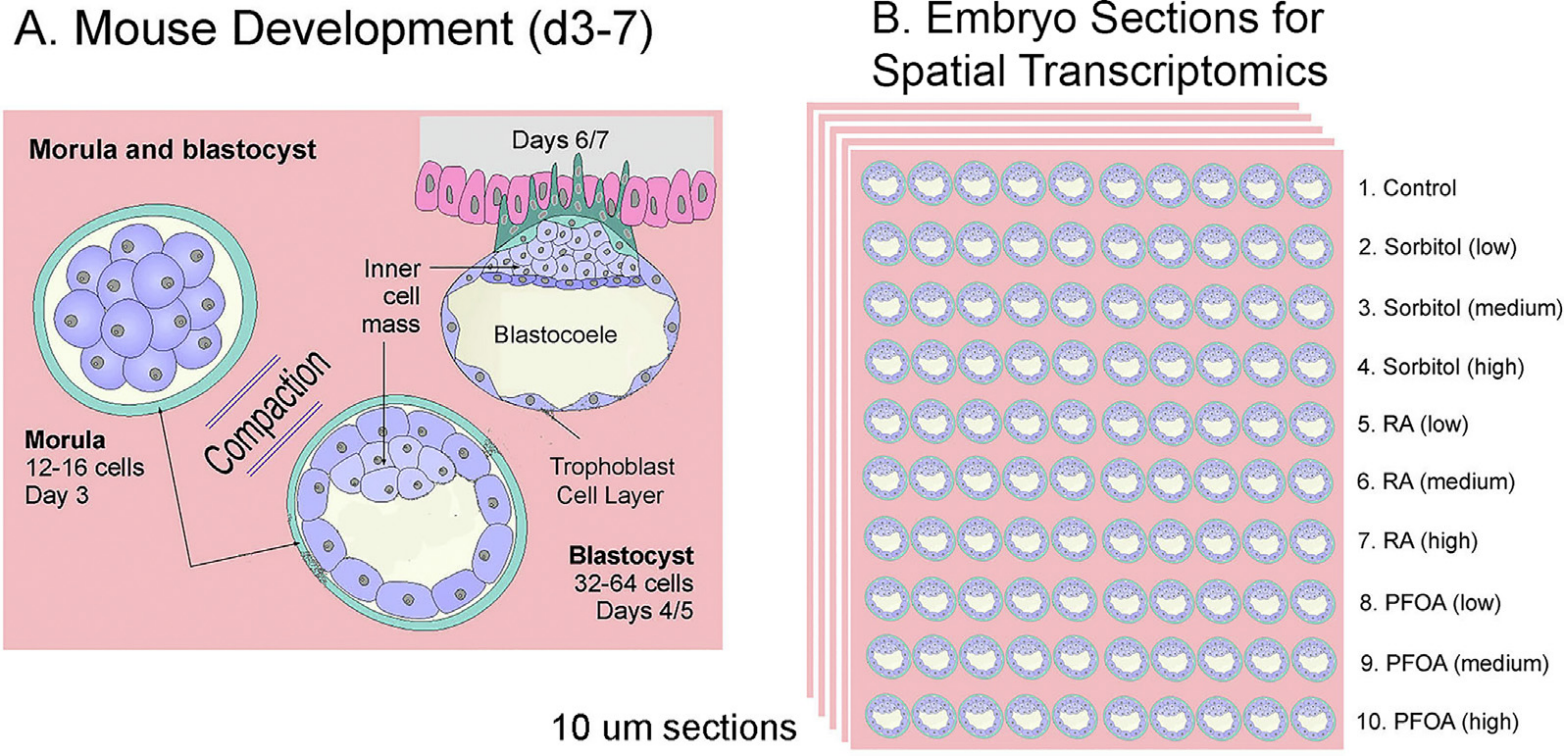
Proposed Embryo Culture Devices for Future Space Embryo Research. **A**, Overview of mouse development from d3-7. **B**, Proposed experimental design for the spatial transcriptomic sections. The embryos are embedded in a collagen matrix to maintain their position. If the embryos develop from the 2-cell stage, the orientations will be random rather than aligned as shown here.

An advantage of the Lei et al.^1^ approach is that using a totally automated system avoids human error and lowers the risk of bacterial contamination, as observed by Wakayama et al.^2^ in all of their experiments. Astronauts must conduct many tasks and it would be difficult to have one trained in advanced embryology. However, a totally automated system does not allow one to prevent unanticipated issues that might arise. For example, the totally automated system contained a camera for time-lapse photograph of the embryos as they developed. Unfortunately, in the experimens by Lei et al.,^1^ the embryos clumped together in space and only a very few embryos along the edges could be visualized. Since Lei et al.^1^ used a totally automated system on an unmanned recoverable satellite, an astronaut could not attempt to shake the incubator or otherwise attempt to reduce the clumping. One possible solution to this issue, would be to mount the 2-cell embryos in a matrix, such as collagen low melting-temperature agarose (Fig. 4). Physically separating the embryos would prevent clumping and allow sectioning for antibody imaging or spatial transcriptomics of a large number of embryos on earth. However, experiments need to be conducted on earth to ensure that embryonic development can proceed normally in such a matrix.

An advantage of the Wakayama et al.^2^ approach is that astronauts could troubleshoot any issues that might arise. For example, when the astronaut applies the solutions to the embryo culturing devices with syringes, they can control how fast or slow the fluids are added to allow proper administration and mixing. While microscopic analyses were not done in space by Wakayama and colleagues on the ISS, future astronauts trained in confocal microscopy, for instance, could potentially make more detailed time-lapse images of developing embryos than could be done with an automated device with a crude microscope as was done by the Lei et al..^1^

The best advice for future space embryo studies is to utilize the best practices learned by both studies. An “embryo in a box” totally automated system on the ISS would avoid requiring extensive training of the astronauts in embryology but would allow astronauts to tweak the device if issues arise in orbit. Also, it would allow the astronaut to move the device to other instruments, such as a confocal microscope. Heavy G-forces on 2-cell embryos in the Lei et al.^1^ studies could adversely affect embryo development. Likewise, freezing and thawing embryos, as done by Wakayama et al.,^2^ can adversely affect development. Perhaps the best strategy would utilize “IVF in a box” totally automated technologies that would allow in vitro fertilization (IVF) of frozen and thawed oocytes and sperm in orbit. This could be followed by a totally automated system that can analyze development in space with intime-lapse confocal microscopy controlled by an astronaut. Astronauts would likely be needed to make sure such complex totally automated systems are running properly, which could not be done with an unmanned recoverable satellite.

## 6 Future perspectives

*The microgravity or the very, very low amount of gravity that we*

*have up in space forces some changes in different processes.*

*It forces changes in us as human beings.*

Laurel Clark, collected essays,^34^

Laurel Clark (1961–1983) was a NASA astronaut, medical doctor, United States Navy captain, and Space Shuttle mission specialist. Tragically, she lost her life, along with her six fellow crew members, in the Space Shuttle Columbia disaster. Posthumously, Clark was honored with the Congressional Space Medal of Honor. The cited excerpt is from her collection of essays, published posthumously.^34^ As an astronaut, she was especially concerned with the issues of human evolution beyond the Earth to ensure the survival of the species.

Further research is imperative to comprehensively understand the effects of microgravity (micro-G) from fertilization to birth in mammalian models, such as mice, to gain insights into potential implications for humans. Utilizing unmanned retrievable satellites presents limitations, particularly in the need for fully automated embryo culturing devices that cannot be adjusted after launch. For future embryo experiments on manned space stations, leveraging the expertise of trained and highly skilled embryologists, well-versed in the intricate details of mouse in vitro fertilization, would be advantageous over relying on untrained astronauts. Skilled embryologists can provide troubleshooting for unforeseen issues and conduct more intricate experiments, such as returning blastocyst embryos to pseudo pregnant mice.

Additionally, given the substantial costs associated with conducting these experiments, a more thorough analysis is warranted than what is offered by whole embryo bulk RNA-seq or bisulfite sequencing. Techniques such as recently developed spatial transcriptomics and spatial epigenomics experiments, where the transcriptome and epigenome can be analyzed at the single-cell level in each blastocyst embryo, should be considered for a more nuanced understanding (Fig. 4). Finally, to better understand the effects of ionizing radiation in space, further studies are needed beyond the double-strand break analyses that were conducted in both space embryo studies. For instance, assays can be done to single-base mutations or small or large DNA rearrangements using both short and long-read whole genome sequencing.

## Funding

This work was supported by an NIH ECHO grant to DMR, UG3 OD023285.

## Ethical approval

This work does not contain any studies with human or animal subjects performed by any of the authors.

## CRediT authorship contribution statement

**Douglas M. Ruden:** Writing – review & editing, Writing – original draft, Supervision, Funding acquisition, Conceptualization. **Daniel A. Rappolee:** Writing – review & editing.

## Declaration of competing interest

The authors declare the following financial interests/personal relationships which may be considered as potential competing interests: Douglas M Ruden reports financial support was provided by 10.13039/100000002National Institutes of Health. If there are other authors, they declare that they have no known competing financial interests or personal relationships that could have appeared to influence the work reported in this paper.
